# Upregulation of microRNA-524-5p enhances the cisplatin sensitivity of gastric cancer cells by modulating proliferation and metastasis via targeting SOX9

**DOI:** 10.18632/oncotarget.13479

**Published:** 2016-11-21

**Authors:** Jing Wang, Xiaofeng Xue, Han Hong, Mingde Qin, Jin Zhou, Qing Sun, Hansi Liang, Ling Gao

**Affiliations:** ^1^ Department of General Surgery, The Second Hospital Affiliated to Jiaxing University, Jiaxing, 314000, Zhejiang Province, P.R. China; ^2^ Department of General Surgery, The First Affiliated Hospital of Soochow University, Suzhou, 215006, Jiangsu Province, P.R. China; ^3^ Department of Hepato-Pancreato-Biliary Surgery, Suzhou Municipal Hospital, The Affiliated Suzhou Hospital of Nanjing Medical University, Suzhou, 215006, Jiangsu Province, P.R. China; ^4^ The Stem Cell and Biomedical Material Key Laboratory of Jiangsu Province (The State Key Laboratory Incubation Base), Soochow University, Suzhou, 215006, Jiangsu Province, P.R. China

**Keywords:** gastric cancer, microRNA, MiR-524-5p, SOX9, chemoresistance

## Abstract

Cisplatin-based chemotherapy is the most commonly used treatment regimen for gastric cancer (GC), however, the resistance to cisplatin represents the key limitation for the therapeutic efficacy. Aberrant expression of MiR-524-5p appears to be involves in tumorigenesis and chemoresistance. However, the mechanism by which miR-524-5p mediates effects of cisplatin treatment in GC remains poorly understood. Expressions of MiR-524-5p was detected in GC tissues and cell lines by qRT-PCR. Cell proliferation was observed by MTT assay; Cell migration was detected by transwell migration and invasion assay. The targeting protein of miR-524-5p was identified by luciferase reporter assay and western blot. We found that downregulation of miR-524-5p in GC tissues and cell lines. SC-M1 and AZ521 cells resistant to cisplatin expressed low levels of miR-524-5p in comparison to the sensitive parental cells. Overexpression of miR-524-5p expression in SC-M1 and AZ521 cells inhibited cell proliferation, migration, and invasion, and conferred sensitivity to cisplatin-resistant GC cells. Subsequently, we identified SOX9 as a functional target protein of miR-524-5p and found that SOX9 overexpression could counteracts the chemosensitizing effects of miR-524-5p. These results provide novel insight into the regulation of GC tumorigenesis and progression by miRNAs. Restoration of miR-524-5p may have therapeutic potential against GC.

## INTRODUCTION

Gastric cancer (GC) is one of the most frequent tumors and the second leading cause of cancer-related death worldwide, with East Asia accounting for more than half of the annual cases [1]. It is estimated that the overall 5 years survival for GC patients is only 20% [2]. The surgery remains the basic treatment for patients with localized GC, however, the majority of patients are diagnosed at an advanced stage [3]. Metastasis is found in more than half of advanced GC patients and causes the majority of cancer-related mortalities [4–5]. Cisplatin is the most widely used first-line chemotherapeutic agent for GC. However, a large number of patients will develop cisplatin resistance which is associated with recurrence and metastasis [6]. Moreover, little improvement in long-term survival has been made in recent years, partly because of the chemotherapeutic drug resistance. Therefore, it is of great importance to find new molecular markers that will help improve the sensitivity of GC cells to cisplatin.

MicroRNAs (miRs) are small noncoding RNAs which regulate gene expression at transcriptional and posttranscriptional levels [7]. Increasing evidence has indicated that miRs have important roles in various biological processes [8–9]. Aberrant expression of miRs is known to be correlated with GC progression. In fact, a number of studies have already begun to seek miRNAs involved in sensitizing or causing resistance to chemotherapy, thus providing potential new targets and mechanisms as optimized treatment options [9–10]. Previous studies have investigated the role of miRNA-524-5p (miR-524-5p) in various types of cancer. Liu et al. reported that miR-524-5p was inversely associated with the activity of MAPK pathway and could inhibit MAPK signaling, tumor proliferation as well as migration in melanoma cells [11]. Chen *et al.* found that miR-524–5p was identified to be associated with overall survival and pathological grade of glioma patients [12]. However, the roles of miR-524-5p in cisplatin resistance for GC and the related mechanisms are still unclear. In this study, we investigated the effect of miR-524-5p on GC and identify its target protein involving chemotherapeutic resistance.

## RESULTS

### MiR-524-5p is downregulated in GC tissues and cell lines

**Figure 1:**
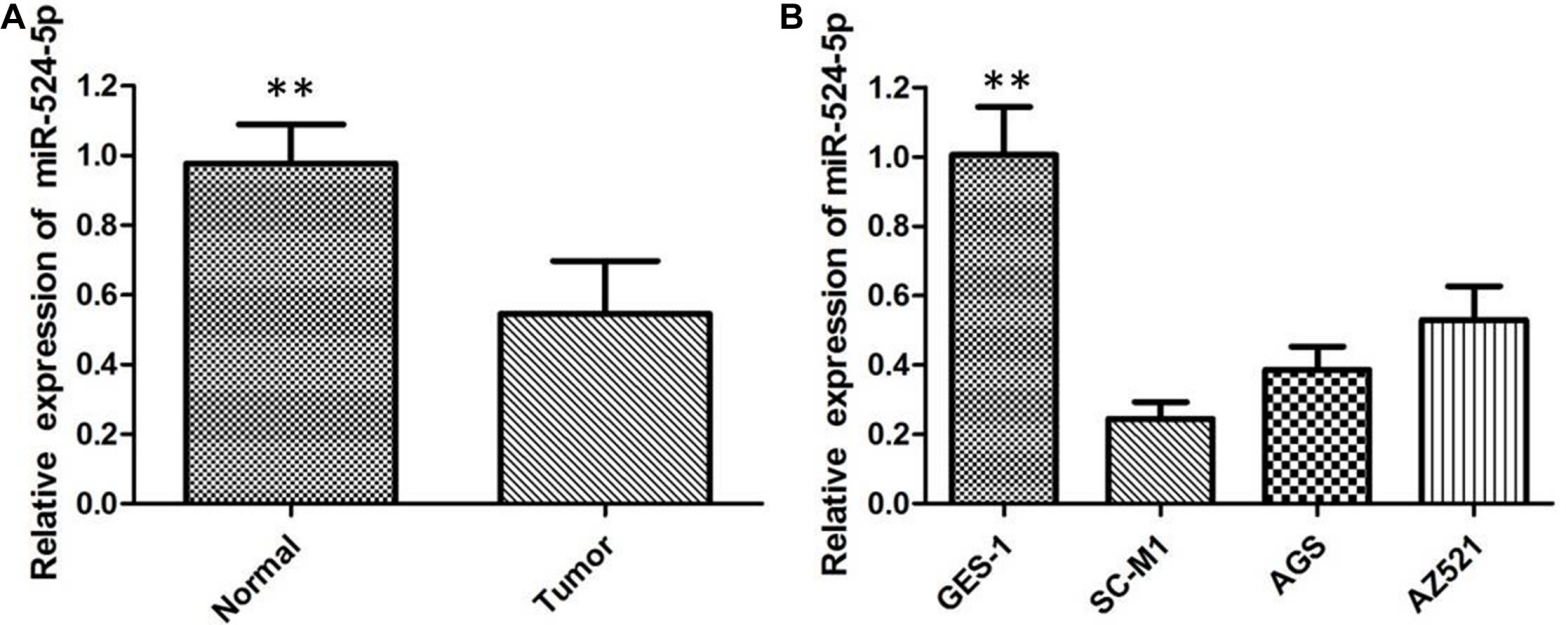
(**A**) The miR-524-5p is down-regulated in GC tissues compared with the corresponding adjacent non- neoplastic tissues; (**B**) The relative expression levels of miR-524-5p in GC cell lines in comparison with human normal gastric epithelial mucosa GES1- cell line

To the best of our knowledge, the present study was the first to assess the expression levels of miR-524-5p in 50 pairs of GC tissues and the adjacent nonneoplastic tissues by qRTPCR analysis. The results revealed that miR-524-5p expression levels in GC tissues were significantly lower compared with those in healthy tissues, and 31/50 samples displayed a reduction of ≤ 50% (Figure 1A). Then we correlated miR-524-5p levels with different clinicopathological factors of GC tissues. We found that low miR-524-5p expression was more frequently detected in GC patients with larger tumor size, positive lymph node metastasis, and advanced TNM stage. These results indicated that miR-524-5p may represent a potential tumor suppressor in GC. When compared with the human normal gastric epithelial mucosa GES1 cells, the expression levels of miR-524-5p were significantly decreased in SC-M1, AGS, and AZ521 cells, indicating that low levels of MiR-524-5p may be relevant to the development of GC (Figure 1B).

### Cisplatin-resistant GC cells have low miR-524-5p expression

**Figure 2:**
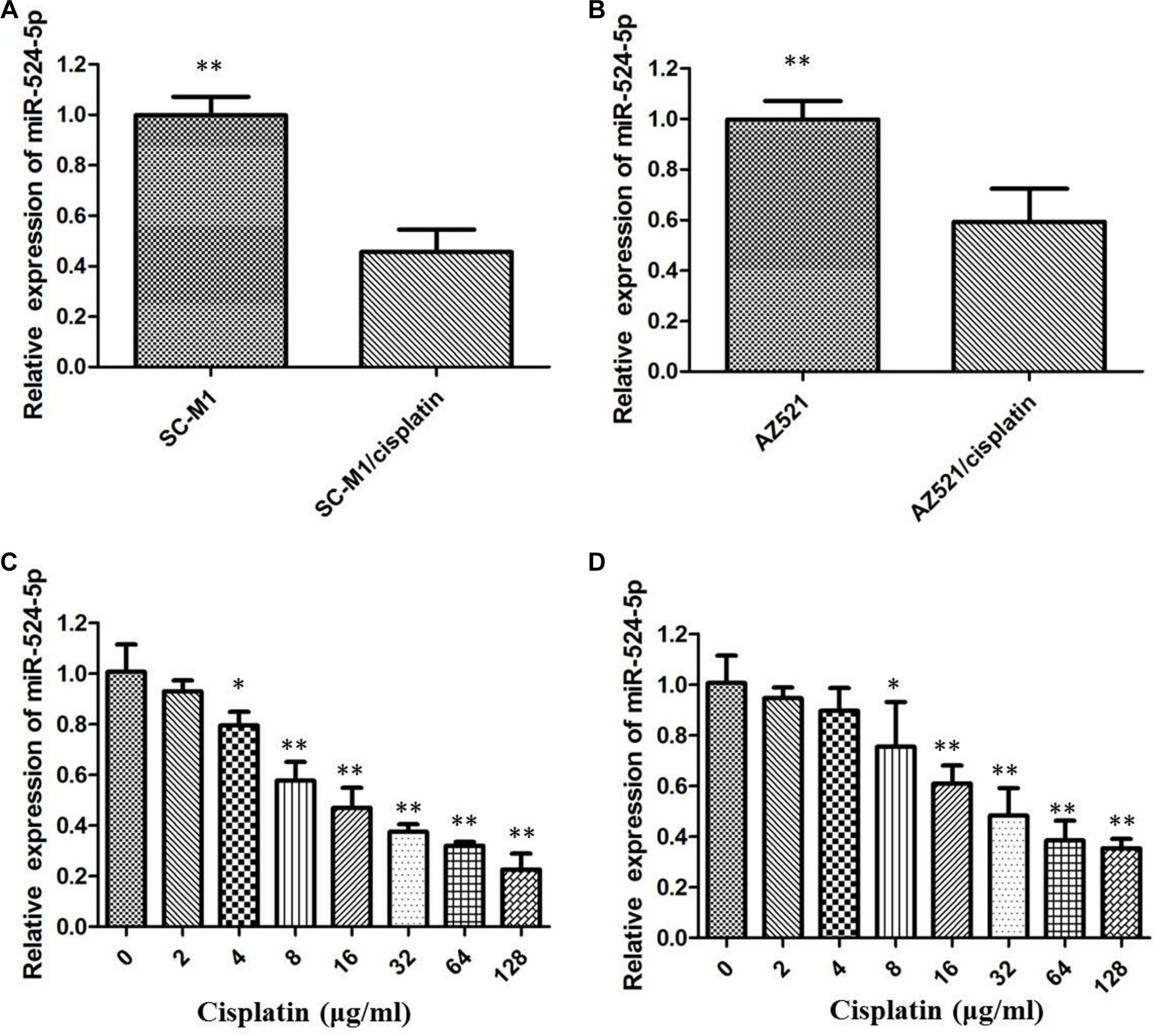
(**A**) Rhe level of miR-524-5p was down-regulated in SC-M1/cisplatin cells compared to the parental cells; (**B**) The level of miR-524-5p was down-regulated in AZ521/cisplatin cells compared to the parental cells; (**C**) The miR-524-5p level was measured by qRT-PCR after different concentration cisplatin treatment in SC-M1 cells; (**D**)The miR-524-5p level was measured by qRT-PCR after different concentration cisplatin treatment in AZ521 cells (*p < 0.05, **p < 0.01=.

To establish cisplatin-resistant GC cells, we repeatedly treated GC cells with increasing concentrations of cisplatin, starting from a low dose. We obtained three lines of cisplatin-resistant GC cells derived from SC-M1 and AZ521 cells. Then IC50 was determined to be 28.85 μg/ml for SC-M1/cisplatin and 17.85 μg/ml for AZ521/cisplatin, both of which were much higher than their parental cells, indicating that cisplatin-resistant GC cells exhibited a significantly decreased sensitivity to cisplatin. We further assessed the expression of miR-524-5p in these cisplatin resistant GC cells. As a result, the level of miR-524-5p was striking down-regulated in SC-M1/cisplatin and AZ521/cisplatin cells compared to their parental cells (Figure 2A and 2B). what's more, the miR-524-5p expression was negatively correlated to the dose of cisplatin. These above resutls reveal that the grade of cisplatin resistance might be associated with miR-524-5p level (Figure 2C and 2D).

### Effects of miR-524-5p on cell proliferation, invasion and migration in GC cells

**Figure 3:**
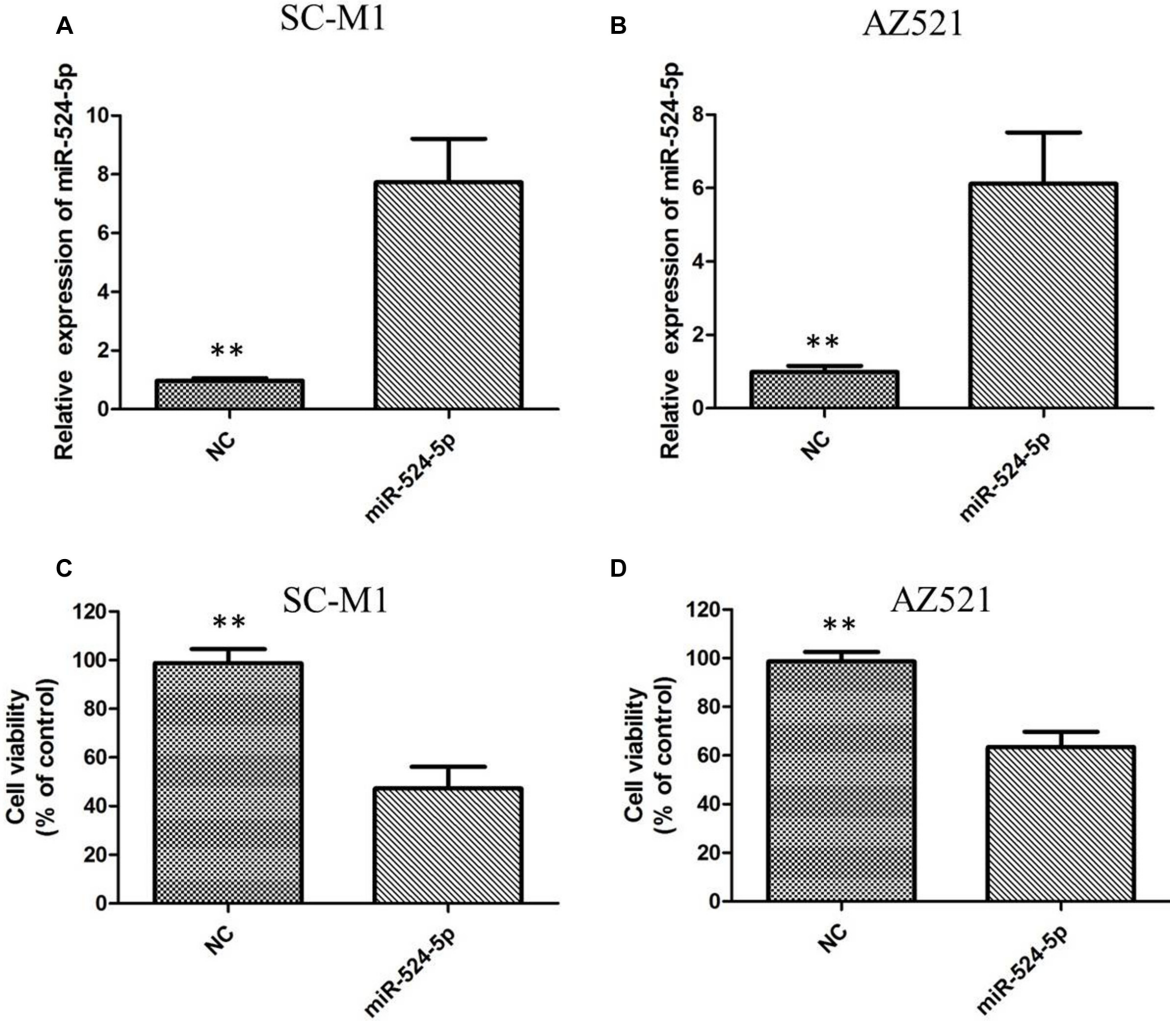
(**A**) Transfection of miR-524-5p mimics into SC-M1 cells increased the expression of miR-524-5p as indicated by qRT-PCR analysis; (**B**) Transfection of miR-524-5p mimics into AZ521 cells increased the expression of miR-524-5p as indicated by qRT-PCR analysis; (**C**) The MTT assay revealed significant inhibition of cell proliferation in SC-M1 cells following miR-524-5p mimics transfection; (**D**) The MTT assay revealed significant inhibition of cell proliferation in AZ521 cells following miR-524-5p mimics transfection

**Figure 4:**
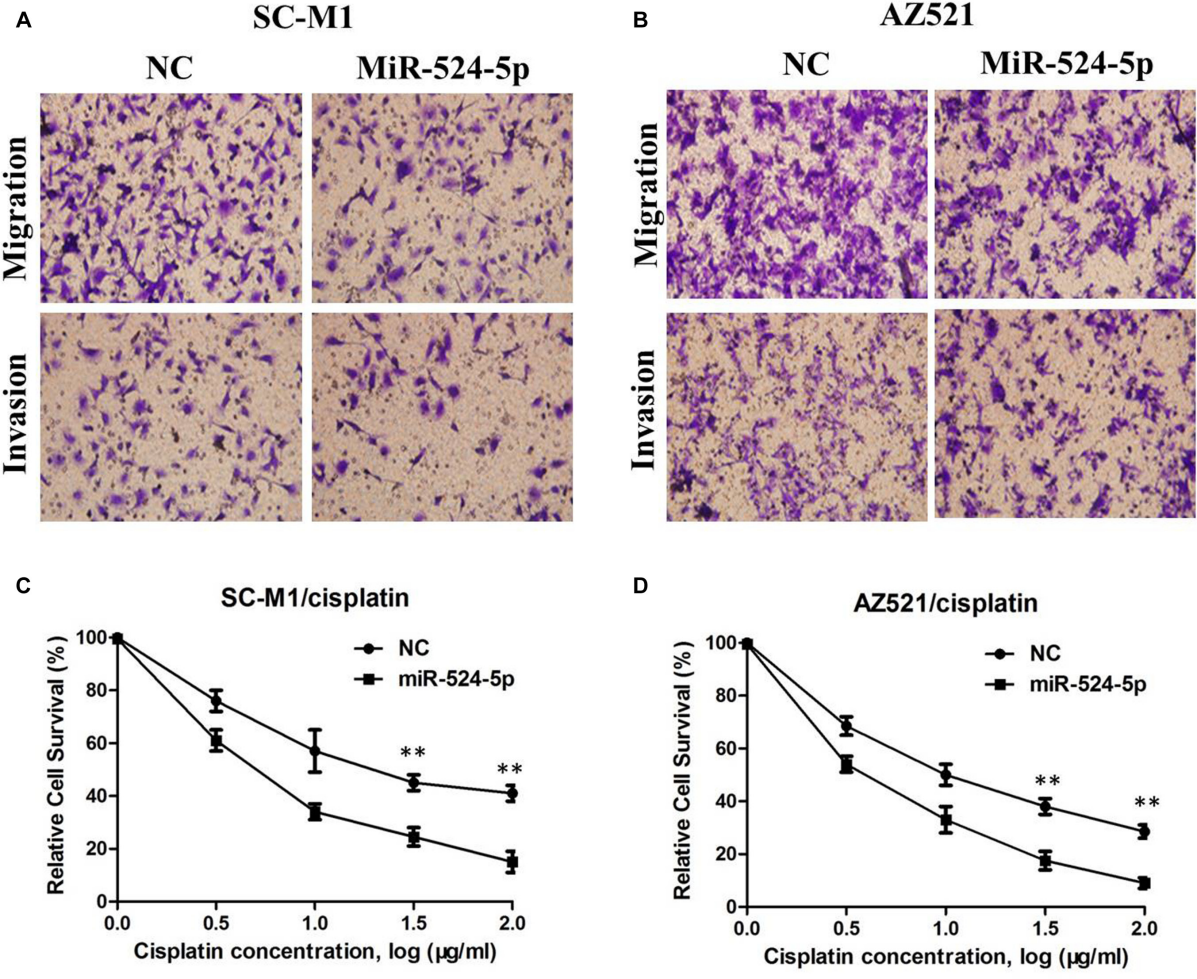
(**A**) The transwell assay showed significant inhibition of cell migration and invasion of SC-M1 cells following miR-524-5p mimics transfection; (**B**) The transwell assay showed significant inhibition of cell migration and invasion of AZ521 cells following miR-524-5p mimics transfection; (**C**) The representative curves of growth inhibition of SC-M1/cisplatin after transfecting miR-524-5p mimics or control; (**D**) The representative curves of growth inhibition of AZ521/cisplatin after transfecting miR-524-5p mimics or control

To investigate the potential effect of miR-524-5p on the progression of GC, we transfected GC cell line SC-M1 and AZ521 cells with either miR-524-5p mimics (miR-524-5p) or negative control miRNA mimics (miR-NC). The miR-524-5p expression was determined using qRT-PCR in SC-M1 and AZ521 cells (Figure 3A and 3B). MTT assay showed that the growth rate of SC-M1 and AZ521 cells with miR-524-5p overexpression was significantly lower than the normal control (Figure 3C and 3D). The role of miR-524-5p on cell migration and invasion, two key determinants of malignant progression and metastasis, was assessed in human GC cell lines by transwell assay. Our results showed that miR-524-5p overexpression significantly suppressed the migration and invasion of SC-M1 and AZ521 cells (*P* < 0.05; Figure 4A and 4B). Taken together, these results suggested that miR-524-5p might participate in SC-M1 and AZ521 cells proliferation and metastasis.

### Increased miR-524-5p level renders chemosensitive to cisplatin in GC cells

To evaluate further whether low miR-524-5p expression is essential for cisplatin resistance, we treated the cisplatin-resistant cells with miR-524-5p mimic and inhibitor. The results revealed that overexpression of miR-524-5p partially increased the cell's sensitivity to cisplatin (Figure 4C and 4D), suggesting that low miR-524-5p expression could contribute to cisplatin resistance in human GC cells.

### MiR-524-5p inhibits SOX9 expression through binding its 3′UTR

**Figure 5:**
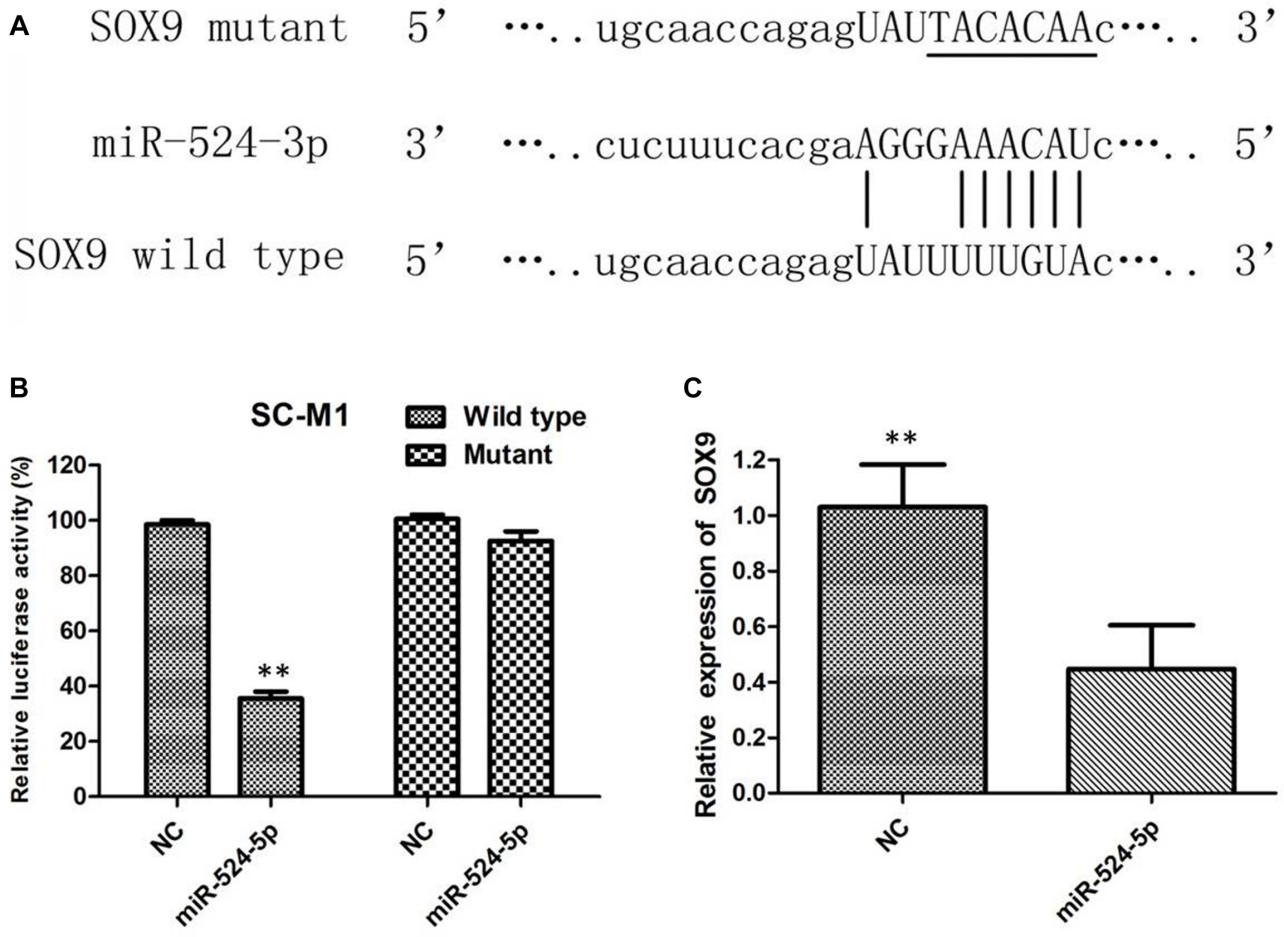
(**A**) The predicted miR-524-5p target site in the wild-type SOX9 3′ UTR and the corresponding mutated 3′ UTR sequence are shown; (**B**) SC-M1 cells were transfected with luciferase construct and the miR-524-5p mimic or the negative control. Relative luciferase activity was measured after 48 h transfection; (**C**) The mRNA levels of SOX9 in SC-M1 were analyzed after miR-524-5p mimics and control tranfection.

The present study aimed to elucidate the underlying mechanism of the tumorsuppressive role of MiR-524-5p in GC. We search for its candidate target genes by bioinformatics analysis. SOX9 has been verified as a functional target of miR-524-5p, as MiR-524-5p efficiently controls SOX9 expression by directly targeting a sequence motif in the coding region of SOX9. To validate our hypothesis, the wild type 3′UTR of SOX9 and its corresponding mutant counterparts were clone into luciferase vector (Figure 5A). Relative luciferase activity assays revealed that the luciferase intensity was suppressed by miR-524-5p, but not in mutant 3′UTR (Figure 5B). To further confirm that SOX9 is a target gene of miR-524-5p, qRTPCR and western blot analysis were used to detect effects of miR-524-5p mimics on the expression of SOX9 in GC cells. The expression of SOX9 was evidently decreased after overexpression of miR-524-5p at the mRNA level compared with that in the negative controltransfected cells (Figure 5C).

### SOX9 regulates proliferation, migration and cisplatin sensitivity of GC cells

**Figure 6:**
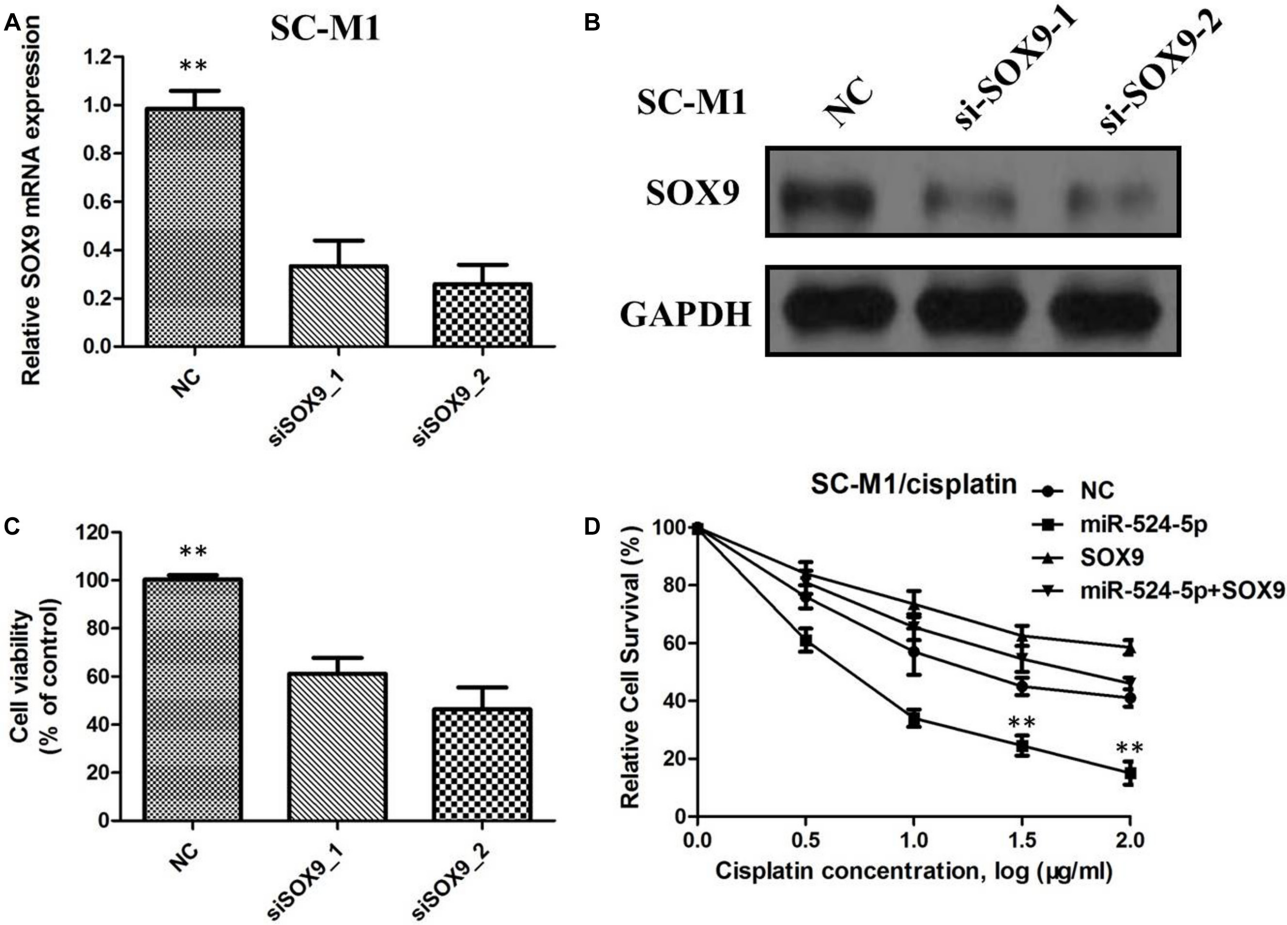
(**A**) The relative SOX9 protein expression level was significantly decreased in siRNA-transfected SC-M1 cells; (**B**) The relative SOX9 protein expression level was significantly decreased in siRNA-transfected SC-M1 cells; (**C**) The growth rate of siRNA-transfected SC-M1 cells was significantly reduced compared with the negative controltransfected group; **(D**)MiR-524-5p mediated cisplatin sensitivity was attenuated after transfection with SOX9-expressing plasmid in SC-M1 cells.

To investigate whether MiR-524-5p exerts its tumorsuppressive function through SOX9, SOX9 was silenced in GC cells using siRNA. The qRTPCR and western blot analyses confirmed that si-SOX9-1 and si-SOX9-2 significantly decreased the mRNA and protein expression of SOX9 in SC-M1 cells (Figure 6A and 6B). Following SOX9 silencing, the growth rate of SC-M1 cells was significantly reduced compared with that in the negative controltransfected group (Figure 6C). Furthermore, after transfection with SOX9-expressing plasmid, SC-M1 cells were treated with different concentration of cisplatin. As shown in Figure 6D, miR-524-5p mediated cisplatin sensitivity was attenuated after transfection with SOX9-expressing plasmid in SC-M1 cells, indicting that the regulatory of miR-524-5p on cisplatin sensitivity through target SOX9 protein in GC.

## DISCUSSION

Over the last few decades, systematic chemotherapy has significantly improved survival of patients with advanced GC patients [13–14]. Cisplatin-based chemotherapy is the most commonly used treatment regimen, however, most patients eventually become insensitive to cisplatin. Thus, acquisition of cisplatin resistance is a major clinical obstacle for treatment of GC. Nevertheless, the molecular basis for in GC remains unclear. Understanding the mechanism of cisplatin resistance is essential for GC treatment.

MiRNAs are a class of short single-stranded RNAs, which suppress gene expression by binding to the 3′-unstranslated region (UTR) of the target mRNA to inhibit translation and/or promote mRNA degradation. Recently, an increasing number of studies demonstrated that miRNAs also target the 5′-UTR and coding region of mRNAs [15–16]. MiRNAs have been involved in various cell processes, including chemoresistance. Although it is clear that dysregulated miRNAs play an important role in the initiation and progression of cancer, acting as tumor suppressors or oncogenes, less has been known concerning the relationship between particular miRNA and GC. Hence, it is of great value to explore the function of miRNAs specifically involved in GC carcinogenesis and dig out the underlying mechanisms.

Dysregulation of miR5245p is a frequent event in melanoma and glioma cells, suggesting that miR5245p may play an important role in tumor progression. Our results showed that miR-524-5p was down-regulated in 31 (31/50, 62%) GC tissues compared with the adjacent tissues and the expression of miR-524-5p in GC tissues was lower than in adjacent tissues. We also found that lower expression of miR-524-5p in GC specimens was correlated with tumor size, invasion depth, and TNM stage. Forced expression of miR-524-5p inhibited cell proliferation and invasion in GC cell lines, indicating that repression of miR-524-5p might promote tumor progression in gastric carcinogenesis. Furthermore, our findings confirmed that resistance to cisplatin in GC cells was associated with the expression of miR-524-5p. Overexpression of miR-524-5p in GC cells enhanced their sensitivity to cisplatin. Finally, we found that miR-524-5p induced cisplatin-resistance improvement mainly depends on the down-regulating SOX9. All of this evidence indicated that miR-524-5p may represent a novel therapeutic target in GC patients.

We next try to identify the molecular mechanism by which miR5245p acts as a suppressor in GC. We used miRNA target prediction programs (Targetscan 6.2 and miRDB) to search candidate targets. Luciferase assay and Western blot analysis showed that miR5245p inhibited the expression of SOX9 by directly targeting its 3′UTR. We also found that enforced expression of miR5245p significantly reduced the mRNA level of SOX9 in GC cells, suggesting that SOX9 was a target of miR5245p in GC cells.

SOX9 belongs to the SOX (SRY-related HMG box) family of transcription factors that acts as an important regulator of several cellular processes [17–18]. Recent studies have shown that SOX9 has important roles in GC development and progression [19]. In this study, our data showed that silencing of SOX9 inhibited the proliferation of GC cells, suggesting that it might function as an oncogene. Moreover, MiR-524-5p induced cisplatin sensitivity depends on the SOX9 regulation. MiR-524-5p mediated cisplatin sensitivity was attenuated after overexpression of SOX9 in GC cells.

In conclusion, our study showed that miR-524-5p is downregulated in GC tissues and cell lines and that low expression of miR-524-5p is associated with lymph-node metastasis and poor pTNM stage. Moreover, enforced expression of miR-524-5p suppressed GC cell proliferation and invasion, and improved cisplatin-resistance of GC cells through directly targeting SOX9. These results provide novel insight into the regulation of gastric tumorigenesis and progression by miRNAs. Restoration of miR-524-5p may have therapeutic potential against GC.

## MATERIALS AND METHODS

### Patients and specimens

Fresh GC tissues and paired non-cancer tissues from patients with GC were collected from The First Affiliated Hospital of Soochow University between 2010 and 2014. Informed consent for the use of samples was obtained from all patients. All samples were diagnosed by 2–3 pathologist blindly. Inclusion criteria were patients with primary GC in I–IV stages, having received surgery as initial treatment modality and having complete clinicopathologic data. Clinicopathologic data included age, sex, histopathologic diagnosis and pathologic tumor stages. Pathologic stage was according to the revised international system. All patients gave informed consent for participation in this study. This study was approved by both the Clinical Research Ethics Committee of Soochow University.

### Cell lines

The human GC cell lines SC-M1, AGS, and AZ521 were obtained from the American Type Culture Collection (Manassas, VA, USA), while the human gastric epithelial mucosa GES1 cell line was purchased from the Shanghai Institutes for Biological Sciences of the Chinese Academy of Sciences (Shanghai, China). The cell lines were cultured in RPMI 1640 medium (Gibco, USA) containing with 10% fetal bovine serum (FBS) (Gibco, USA), 100 U/ml penicillin, and 100 mg/ml streptomycin at 37°C.

### Plasmids and cell transfection

A miR5245p mimic, SOX9-expressing plasmid, siRNA- SOX9, and the corresponding negative controls were purchased from Shanghai GenePharma Co., Ltd. (Shanghai, China). Cells were seeded in 6well plates at 30% confluence one day prior to transfection. The cells were transfected using Invitrogen Lipofectamine^®^ 2000 (Thermo Fisher Scientific, Inc.), according to the manufacturer's protocol.

### Luciferase reporter assay

The putative miR-524-5p binding sequences from the 3′-UTR of SOX9 mRNAs were cloned downstream of a CMV promoter-driven firefly luciferase open reading frame in a pMir-Report vector (Applied Biosystems, Carlsbad, CA, USA). Mutant versions of the 3′UTR were also generated using standard PCR based protocols. For luciferase reporter assay, cells transfecting either miR-524-5p mimics or miR-NC were plated in a 24-well plate, and then transfected with wild type or mutant luciferase constructs using Lipofectamine 2000 (Invitrogen, Carlsbad, CA, USA). Cells were collected 48 h after transfection and analyzed using the Dual-Luciferase reporter Assay System (Promega, Madison, WI, USA) following manufacturer's instruction.

### qRT–PCR

Total RNA was extracted from the cells (or tumor tissues) using mirVana™ miRNA Isolation Kit (Ambion) according to the manufacturer's protocol. miR-524-5p and RNU48 cDNA were synthesized by TaqMan PreAmp Master Mix Kit and TaqMan MicroRNA Assays (Applied Biosystems) according to the manufacturer's instructions to quantify microRNA expression. Relative expression of target microRNAs was calculated using the ^ΔΔ^Ct method and normalized to RNU48.

### Western blot

Cells were washed with PBS for twice, scraped off the culture dishes, treated with 200 μl RIPA buffer (Sigma) containing protease inhibitors and were then transferred to 1.5-ml tubes. The tubes were kept on ice for 30 min for cell lysed. After a centrifugation at 13500 rpm for 15 min under 4°C, the supernatants were collected and were subsequently denatured with 2 X SDS protein loading buffer (Tiangen, Beijing, China) at 100°C. Proteins were loaded on 10% SDS-PAGE gels and transferred onto PVDF membranes. Membranes were blocked with 5% BSA in PBS at room temperature for 2 h and incubated with primary antibodies against corresponding antibodies 4°C overnight. The band was detected using HRP-conjugated secondary antibodies along with an enzyme-linked chemiluminescence kit (Beyotime Institute of Biotechnology, Shanghai, China).

### Cell proliferation assay

Cells were transfected with 10 nM miRNA or siRNA by reverse transfection and seeded in 96well plates at 3 × 103 cells per well. After 72 h of incubation, cell proliferation was determined using an MTT assay (Invitrogen) according to the manufacturer's protocol. The number of cells per well was determined by measuring the absorbance at 540 nm. All experiments were performed in triplicate.

### Transwell cell migration assay

A cell migration assay was performed using transwell chambers with a pore size of 0.8 μm. A total of 1 × 10^5^ cells were seeded in serum-free medium in the upper chamber, while medium containing 10% FBS was added as a chemoattractant to the lower chamber. After incubating for 48 h at 37°C, the cells in the upper chamber were carefully removed with a cotton swab, and the cells that had migrated to the reverse face of the membrane were fixed in methanol, stained with Giemsa, and counted.

### Statistical analysis

Each experiment was repeated at least three times. Bands from Western blots were quantified with Quantity One software (Bio-Rad, Hercules, CA, USA). Relative protein and mRNA levels were calculated in comparison to internal b-actin or GAPDH standards. Numerical data are presented as means ± SD. The difference between means was analyzed with ANOVA. Differences were considered significant when *P* < 0.05

## References

[1] Siegel RL, Miller KD, Jemail A (2015). Cancer statistics, 2015. Cancer J Clin.

[2] Cunningham D, Allum WH, Stenning SP, Thompson JN, Van DE Velde CJ, Nicolson M, Scarffe JH, Lofts FJ, Falk SJ, Iveson TJ, Smith DB, Langley RE (2006). Perioperative chemotherapy versus surgery alone for resectable gastroesophageal cancer. N Engl J Med.

[3] Compare D, Rocco A, Nardone G (2010). Risk factors in gastric cancer. Eur Rev Med Pharmacol Sci.

[4] Huang H, Han Y, Zhang C, Wu J, Feng J, Qu L, Shou C (2016). HNRNPC as a candidate biomarker for chemoresistance in gastric cancer. Tumour Biol.

[5] Li Y, Gong J, Zhang Q, Lu Z, Gao J, Li Y, Cao Y, Shen L (2016). Dynamic monitoring of circulating tumour cells to evaluate therapeutic efficacy in advanced gastric cancer. Br J Cancer.

[6] Zhang Z, Kong Y, Yang W, Ma F, Zhang Y, Ji S, Ma EM, Liu H, Chen Y, Hua Y (2016). Upregulation of microRNA-34a enhances the DDP sensitivity of gastric cancer cells by modulating proliferation and apoptosis via targeting MET. Oncol Rep.

[7] Zhang B, Ji S, Ma F, Ma Q, Lu X, Chen X (2016). MiR-489 acts as a tumor suppressor in human gastric cancer by targeting PROX1. Am J Cancer Res.

[8] Qian K, Mao B, Zhang W, Chen H (2016). MicroRNA-561 inhibits gastric cancercell proliferation and invasion by downregulating c-Myc expression. Am J Transl Res.

[9] Huang M, Wu L, Qin Y, Li Z, Luo S, Qin H, Yang Y, Chen J (2016). Anti-proliferative role and prognostic implication of miR-141 in gastric cancer. Am J Transl Res.

[10] Zhou X, Ji G, Chen H, Jin W, Yin C, Zhang G (2015). linical role of circulating miR-223 as a novel biomarker in early diagnosis of cancer patients. Int J Clin Exp Med.

[11] Iu SM, Lu J, Lee HC, Chung FH, Ma N (2014). miR-524-5p suppresses the growth of oncogenic BRAF melanoma by targeting BRAF and ERK2. Oncotarget.

[12] Chen L, Zhang W, Yan W, Han L, Zhang K, Shi Z (2012). The putative tumor suppressor miR-524–5p directly targets Jagged-1 and Hes-1 in glioma. Carcinogenesis.

[13] Holohan C, Van Schaeybroeck S, Longley DB, Johnston PG (2013). Cancer drug resistance: an evolving paradigm. Nat Rev Cancer.

[14] Siddik ZH (2003). Cisplatin: mode of cytotoxic action and molecular basis of resistance. Oncogene.

[15] Ambros V (2004). The functions of animal microRNAs. Nature.

[16] Bartel DP (2004). MicroRNAs: genomics, biogenesis, mechanism, and function. Cell.

[17] Kopp JL, von Figura G, Mayes E, Liu FF, Dubois CL, Morris JPt (2012). Identification of Sox9-dependent acinar-to-ductal reprogramming as the principal mechanism for initiation of pancreatic ductal adenocarcinoma. Cancer Cell.

[18] Thomsen MK, Ambroisine L, Wynn S, Cheah KS, Foster CS, Fisher G (2010). SOX9 elevation in the prostate promotes proliferation and cooperates with PTEN loss to drive tumor formation. Cancer Res.

[19] Sun M, Uozaki H, Hino R, Kunita A, Shinozaki A, Ushiku T (2012). SOX9 expression and its methylation status in gastric cancer. Virchows Arch.

